# Evaluating the translational value of postmortem brain reperfusion technology

**DOI:** 10.1515/tnsci-2020-0179

**Published:** 2021-07-09

**Authors:** Michael Nair-Collins

**Affiliations:** Department of Behavioral Sciences and Social Medicine, Florida State University College of Medicine, Tallahassee, Florida, United States of America

**Keywords:** brain ischemia, brain death, brain perfusion, *Sus scrofa domesticus*, neuroresuscitation, neurorehabilitation

## Abstract

A novel pulsatile-perfusion technology, dubbed Brain*Ex*, has been shown to restore microcirculation and cellular functions in the pig brain, 4 h postmortem. This technology has generated enthusiasm for its translational value for human neuroresuscitation. I offer a critical analysis of the study and its methodology, providing several reasons for skepticism. This includes: all phenomena were observed at different degrees of hypothermia; the physiological and biochemical milieu of the experimental preparation is radically different than the clinical setting of hypoxic-ischemic brain injury; and the study is confounded by uncontrolled traumatic brain injury and lifelong stress in all the animals.

## 1 Introduction

Researchers have developed a novel extracorporeal pulsatile-perfusion technology along with a novel perfusate, collectively dubbed “Brain*Ex*,” which has been shown to restore microcirculation, along with specific molecular and cellular functions of the large mammalian brain, 4 h postmortem [1]. Restoration of brain circulation and cellular functions was maintained for a period of up to 6 h. This technology appears to demonstrate far greater tolerance of ischemia and anoxia in the large mammalian brain than is currently recognized, and thus may have potential for eventual translation into neurocritical care settings.

The Brain*Ex* platform consists of a complicated array of components. First, the surgical procedure involved isolating the brain and vascular supply above the medulla oblongata, with a perfusion device connected to the carotid arteries. The perfusion device allowed pulsatile waveforms that can mimic physiological waveforms (i.e., the heartbeat) and incorporated both gas infusion and hemodiafiltration mechanisms. Essentially, this is like a combination of an extracorporeal membrane oxygenation device and a dialysis machine. The perfusate was hemoglobin-based and acellular, with echogenic particles allowing measurement of perfusion dynamics using an ultrasonogram. The perfusate also contained anticoagulative and cytoprotective (antiapoptotic and anti-necrotic) agents [1].

The study, published in *Nature* and accompanied by invited commentaries, has generated quite a bit of optimism and enthusiastic commentary regarding not only its potential for basic science research, but also for translational research involving human neuroresuscitation. Lead author Vrselja and colleagues wrote,

We have shown that microcirculation and specific molecular and cellular functions in the large mammalian brain can be restored under *ex vivo* normothermic conditions after an extended [post-mortem interval]… Perhaps most importantly, with the appropriate intervention, the mammalian brain retains a greater capacity for metabolic and neurophysiologic resilience to anoxic or ischaemic insult than is currently appreciated [1, p. 342].

Furthermore, “This experimental approach…could potentially help to bridge the gap between basic neuroscience and clinical research, especially as it pertains to the human brain” [1, p. 342].

Going much further in their optimism for the potential translational value of the technology, Stuart Youngner and Insoo Hyun suggested that,

If technologies similar to Brain*Ex* are improved and developed for use in humans, people who are declared brain dead (especially those with brain injuries resulting from a lack of oxygen) could become candidates for brain resuscitation rather than organ donation [2, p. 302–303].

Farahany and colleagues, commenting on the study that they described as “remarkable” [3, p. 299], suggested that this study “opens up possibilities that were previously unthinkable” [3, p. 300].

It would seem from these and other commentaries that this study and technology have opened a fruitful new line of research for human neuroresuscitation, and in dramatic fashion no less. In light of its high profile, and the enthusiastic evaluations of its translational value, the study and methodology deserve a careful, critical analysis.

## 2 Critical analysis

### 2.1 Hypothermia

The study has been described many times as occurring “at normal body temperature” [e.g., 2, p. 304]. Vrselja and colleagues stated that “functions in the large mammalian brain can be restored under *ex vivo* normothermic conditions” [1, p. 342]. However, the assertion that the observed metabolic and other activities took place under normothermic conditions is inaccurate, and correcting this inaccuracy is important for evaluating the scientific and clinical implications of the study. A brief review of the methodology with an emphasis on temperature is appropriate.

The research animals used in the study, about 300 pigs, were obtained from a slaughterhouse. The researchers were present at the slaughterhouse when the pigs were killed by exsanguination and were then decapitated. After the animals were decapitated, the researchers quickly performed some basic procedures to prepare the head for subsequent study, including isolating the major arteries and attaching catheters to them. “In total, these procedures required approximately 10 min to complete, thereby subjecting brains to this length of warm anoxia” [1, p. 344].

The vasculature was then flushed with eight liters of cold (20°C) heparinized saline in a 30 min, three-step procedure involving gravity flush, then a powered closed-loop flow-driven flush, followed by a second gravity flush, to clear the vasculature while rapidly cooling the brains [1, p. 344]. Hence, blood and other fluids, and especially toxic by-products of ischemic anoxia, cytotoxic edema, and cellular necrosis, as well as thrombi – all predictable outcomes of exsanguination plus the 10 min of warm ischemia during the initial procedures – were quickly washed out of the vasculature, and the brains were rapidly cooled using the cold flush.

After the cold flush, the heads were put on ice and transported to the Virginia Tech lab nearby [4]. Apart from 30 min of exposure to room temperature during a craniectomy at the lab, the heads remained on ice for 4 h. After the craniectomy, epidural temperature was 12–15°C [1, p. 344]. When the experiment began, 4 h after decapitation, the brains were rewarmed at a rate of 6°C per hour, from 25 to 37°C [1, p. 344]. This temperature was presumably measured deeper within the brain than the epidural surface, whose temperature was 10–13°C colder than 25°C, as described above; however, the site of measurement during the experiment is not described. The brains reached maximum temperature of 37°C, 2 h into the experiment. The brains were not at any point rewarmed beyond 37°C.

Critically, normal body temperature is species-specific. While 37°C is normothermic for a human, it is hypothermic for the animals used in the study (*Sus scrofa domesticus*), whose normal core body temperature ranges from approximately 38 to 40°C, with the mean approximately 39°C [5,6,7].

In other words, the brains were rapidly flushed of blood and endogenous toxins and cooled with an anticoagulant fluid, then placed on ice for several hours, during which they remained profoundly hypothermic. When the study began, the brains were severely hypothermic; 2 h into the study, the brains reached maximum temperature of 37°C, which is mild hypothermia. But *at no time at all* did the pig brains actually reach normothermia *for pigs*.

Furthermore, normal core body temperature for mammals is cooler than normal brain temperature, due to the high metabolic activity in the brain. For example, one review comparing brain temperature to core temperature in humans found that brain temperature was higher on all measures of core temperature (rectal, blood, etc.) in all 15 studies reviewed; this difference ranged from a mean of 0.39 to 2.5°C [8; see also 9].

Therefore, the phenomena observed by Vrselja and colleagues [1] were all observed at different degrees of hypothermia, but never at normal core body temperature. Notably, it has been known for centuries that hypothermia protects brain tissue.

### 2.2 Physiological and biochemical milieu: experimental preparation versus clinical setting

The cold saline flush is scientifically and clinically important for a second reason: It renders the physiologic conditions in the animals’ brains dramatically different from a real, clinical setting of human anoxic brain injury. In a clinical setting, ischemia-hypoxia causes metabolic acidosis, as lactic acid builds up from the process of anaerobic glycolysis. In turn, brain acidosis, as well as tissue injury, stimulates release of inflammatory cytokines, such as interleukin-6 (IL-6) and tumor necrosis factor alpha (TNFα), thereby triggering systemic inflammation as well as the activation of coagulation pathways, which can cause a variety of systemic coagulopathies [10,11,12,13]. Furthermore, due to decreased availability of energy (via decreased production of adenosine triphosphate, or ATP), the sodium-potassium pump becomes dysfunctional, leading to extrusion of potassium from the cell and intrusion of sodium and water, along with calcium ions, leading to cytotoxic edema [14, p. 23], cell membrane damage or rupture, and cellular necrosis; and all of these factors increase brain swelling and thereby also increase intracranial pressure [12,13]. If severe enough, this can result in brain herniation [15].

These physiologic derangements in the brain also have cascading and reciprocal effects on the rest of the organism, such as systemic inflammation which in turn can exacerbate brain injury; liver damage from coagulopathies can exacerbate brain injury via an accumulation of ammonia in the nervous system [10, p. 800–801]; “catecholamine storm” can occur, which involves unopposed release of massive amounts of sympathetic, stimulant hormones that cause tissue injury including to the myocardium [16,17]; dysfunction of hypothalamic areas results in multiple neuroendocrine derangements which also alter brain chemistry [13]; and so on.

To summarize, the physiologic consequences of lack of blood and oxygen to the brain include a large variety of endogenous toxins, which themselves cause further damage in a cascading and reciprocal fashion, both to the brain itself and in a systemic fashion to the organism as a whole. By immediately washing out these toxins from the pigs’ brains, and by washing out the blood and its products of coagulation, the biochemical, physiologic conditions of the experimental animals’ brains are *radically* different from a real clinical setting.

Finally, the fact that the animals were decapitated is also physiologically relevant, since clinical medicine must address the human organism as a whole, including the cascading and reciprocal effects throughout the organism of a variety of pathological conditions that can be induced by hypoxic-ischemic brain injury.

### 2.3 Confounds: traumatic brain injury and lifelong stress

Following standard slaughtering procedures, all of the research animals were stunned with a combination electric-mechanical stun device that strikes them on the head. The purpose of this is to render the animal unconscious before hanging it upside down for death by exsanguination by severing carotid arteries. However, the stun method is known to be not uniformly effective, and some animals remain conscious [18]. This is important from a scientific perspective for two reasons.

First, all of the research animals were struck with a device that, by design, causes traumatic brain injury. This is itself an important confound in a study of brain function. Second, since the device does not always work, or at least does not always work the same way, we do not know that the traumatic brain injury caused to the pigs was uniform across subjects, and this can be the case even when the animal is stunned to unconsciousness, because there could be different degrees of neurological trauma that cause unconsciousness, and there could be small but meaningful differences in the angle at which the stunner hits the animal, leading to differences in areas and degree of brain trauma.

As a scientific principle, all the animals should be as similar to each other as possible, and especially, relevant confounds need to be avoided or addressed in some way. In a study of brain function, traumatic brain injury in all the research animals is an important confound; compounding this problem, different degrees or manifestations of that brain injury may render the animals’ brains relevantly different from each other; and there is no way to rule this out. This violates the basic scientific principle of using relevantly similar animal subjects to make generalizable conclusions.

Finally, as livestock, the animals were subject to intense physical and psychological stress throughout their brief lives, including being removed early from their mothers, extreme confinement, and cutting off tails and pulling out teeth without anesthesia or other pain management; highly confined and stressful transportation to the slaughterhouse; and the environment of the slaughterhouse itself, with sights, sounds, and smells of blood and death [18,19,20]. This intense stress has well-known impacts on physiology, including on the hypothalamic-pituitary-adrenal axis, the hypothalamic-pituitary-gonadal axis, sympathetic upregulation with consequences for cardiovascular and other functions, immune system dysregulation, and others [20,21]. These stressful conditions, again, were uncontrolled, and thus generate further confounding conditions.

## 3 Discussion

The Brain*Ex* technology has generated a great deal of interest and optimistic commentary, with the hopes that it could be relevant to human neuroresuscitation. In this brief analysis, I have offered several reasons for a more skeptical and measured interpretation of the translational value of this study and its technology.

Specifically, the animals’ brains were hypothermic throughout the entirety of the study. The heparinized saline flush removed endogenous toxins, products of coagulation, and blood, rendering the biochemical environment of the experimental preparation radically different than that of a human patient with anoxic-ischemic brain injury. Finally, the study is significantly marred by the fact that all animals endured intense levels of stress throughout their lives; all animals were struck with a device that, by design, causes traumatic brain injury; and both of these factors were neither controlled nor uniform, thus creating significant confounds in the interpretation of the results.

In consideration of this study’s translational value for human clinical medicine, a measured and skeptical approach is warranted.
